# Recombinant Vaccines against *T. gondii*: Comparison between Homologous and Heterologous Vaccination Protocols Using Two Viral Vectors Expressing SAG1

**DOI:** 10.1371/journal.pone.0063201

**Published:** 2013-05-15

**Authors:** Érica Araújo Mendes, Flavio G. Fonseca, Bárbara M. Casério, Janaína P. Colina, Ricardo Tostes Gazzinelli, Braulia C. Caetano

**Affiliations:** 1 Departamento de Bioquímica e Imunologia, Instituto de Ciências Biológicas, Universidade Federal de Minas Gerais, Belo Horizonte, Brazil; 2 Departamento de Microbiologia, Instituto de Ciências Biológicas, Universidade Federal de Minas Gerais, Belo Horizonte, Brazil; 3 Centro de Pesquisas René Rachou, Fundação Oswaldo Cruz, Belo Horizonte, Brazil; 4 Division of Infectious Disease and Immunology, University of Massachusetts Medical School, Worcester, Massachusetts, United States of America; Federal University of São Paulo, Brazil

## Abstract

The use of recombinant viral vectors expressing *T. gondii* antigens is a safe and efficient approach to induce immune response against the parasite and a valuable tool for vaccine development. We have previously protected mice from toxoplasmosis by immunizing the animals with an adenovirus expressing the protein SAG1 (AdSAG1) of *T. gondii*. We are now looking for ways to improve the vaccination strategy and enhance protection. One limitation of homologous vaccinations (sequential doses of the same vector) is induction of anti-vector immune response that blocks cell transduction, restricts transgene expression and, consequently, compromises the overall outcome of vaccination. One way to avert the effects of anti-vector response is to use different viruses in prime and boost (heterologous vaccination). Bearing this in mind, we generated a modified Vaccinia Virus Ankara encoding SAG1 (MVASAG1), to be tested as boost agent after prime with AdSAG1. Although minor differences were observed in the magnitude of the anti-SAG1 immune response induced by each vaccination protocol, the heterologous immunization with AdSAG1 followed by MVASAG1 resulted in improved capacity to control brain cyst formation in a model of chronic toxoplasmosis in C57BL/6 mice.

## Introduction

*Toxoplasma gondii* is an obligate intracellular protozoan that belongs to Phylum Apicomplexa. The parasite has a heteroxenous life cycle, with different warm blood species, including humans, serving as intermediate hosts that sustain replication of its asexual forms (tachyzoites and bradyzoites). Domestic and wild felines are the definitive hosts that develop sexual stages of the parasite in the gut, and shed infective forms (sporulated oocysts) in feces [1]. Infection of all types of hosts may occur through consumption of meat and viscera of animals infected with *T. gondii*, or through ingestion of water/vegetables contaminated by sporulated oocysts [2]. In both intermediate and definitive hosts, infection is followed by a short period of intense replication of tachyzoites, which culminates with rupture of infected cells and parasite spreading from the initial infection site. The tachyzoites later convert into slow-replicating bradyzoites that become enclosed in intracellular cysts. This stage conversion characterizes the chronic form of toxoplasmosis, which can persist through the entire life of the host [3]. The host immune response plays a major role in the control of tachyzoite replication and stage conversion. On the other hand, parasites use cysts to evade complete clearance by the immune system. Immunocompetent hosts develop a strong and long-lasting Th1-biased response, with production of IL-12, IL-18, TNF-α, IFN-γ, as well activation of T CD4^+^ and T CD8^+^ cells that are essential for survival after infection [4], [5]. The contribution of the humoral immune response to control of toxoplasmosis is less well understood. There are studies suggesting an important role for antibodies during acute phase of infection [6], and others pointing for a minor influence of immunoglobulins in parasite control during chronic disease [7].

Healthy adult humans usually develop a non-symptomatic chronic form toxoplasmosis. However, immune-compromised individuals such as AIDS patients and persons undergoing cancer treatment may present severe forms of the disease, such as encephalitis and pneumonitis. Another risk group consists of children infected during pregnancy. Depending on the time of exposure of the fetus, the consequences may vary from abortion to retinochoroiditis, mental retardation and intracranial calcifications in the surviving infants [8], [9]. Approaches to prevent exposure to *T. gondii* have not been sufficient to reduce infection and the occurrence of toxoplasmosis. Moreover, there is not an effective treatment against the chronic form of the disease, as the available drugs, like sulfadiazine, act only on the proliferative tachyzoites and have no effect on encysted bradyzoites. Thus, the development of vaccines against *T. gondii* is an important alternative for disease control [10], [11].

A great number of immunization strategies have been tested against *T. gondii*, including vaccines based on attenuated parasites, proteins purified from parasites, recombinant proteins and DNA. These immunogens conferred different levels of protection, which depended on type of antigen, the delivery system and the presence of adjuvant in the vaccine formulation. Some of these vaccine candidates have limited perspectives to reach use in humans, as they contain components that are potentially hazardous, such as live parasites or adjuvants that may cause inflammatory side effects (reviewed in [12]).

Among the different approaches for development of more immunogenic vaccines against protozoan parasites, the use of recombinant viral vectors is an alternative of great potential. Viral vectors typically elicit efficient expression of the foreign antigens they encode, which facilitates the presentation and development of specific immune response against the recombinant antigen [13], [14]. The viral vectors were also demonstrated to activate innate immune mechanisms that exert adjuvant effects that improve the immune response against the recombinant product [15]–[18]. The recombinant adenovirus is a well-characterized viral vector that has been used to express antigens from a number of infectious agents and has been safely tested in various immunization protocols with a wide range of hosts, including humans, resulting in different degrees of immune response and protection [19]–[24].

One limitation of the viral vectors, as for most vaccination approaches involving recombinant antigens, is the fact that generation of protective immune response requires multiple exposure of the immune system to the recombinant antigen. Thus, the viral vectors such as the adenovirus are normally used in the so-called prime and boost vaccination protocols [25], [26]. One potential problem of sequential immunizations with the same type of viral vector (homologous prime and boost) is that prime-immunization with a vector may result in production of neutralizing antibodies that could interfere in the transduction of cells in the following rounds of immunization [27], [28]. Therefore, the expression and presentation of the recombinant antigens may be compromised, and so the activation of immune response. In an attempt to circumvent vector-specific neutralizing antibodies and increase the potency of the immunization, many groups have used heterologous vaccination protocols, which comprise different viral vectors carrying the same antigen [29]–[31].

The modified Vaccinia virus Ankara (MVA) was obtained through serial passages of Vaccinia virus in primary chicken embryo fibroblasts [32]. The resulting virus was a replication-deficient and highly attenuated strain, which historically was employed as safer vaccine alternative against smallpox [33]. In time, the high safety profile and the ability to induce intense expression of foreign genes have made MVA a prime candidate for vaccine vector [34]–[37]. Moreover, it was demonstrated that the defect in the morphogenetic program that rendered the MVA incapable of replicating does not alter the expression of recombinant antigens in mammalian cells compared to replication-competent Vaccinia virus [38].

Over the past decade, our group has been engaged in the development of a vaccine against toxoplasmosis based on recombinant adenoviruses encoding the sequences of surface antigens SAG1, SAG2 and SAG3 of *T. gondii*. These are three conserved and abundant antigens of the tachyzoite surface [39], which are believed to mediate parasite attachment to host cells during the process of invasion [40]. Individuals naturally infected with *T. gondii* produce antibodies against these antigens [41]. Also, reports indicate that these antigens have epitopes that are presented in the context of different haplotypes of human histocompatibility complex and are therefore recognized by CD4^+^ and CD8^+^ T cells [42]. We believe that these properties make the SAG antigens suitable candidates for a toxoplasmosis vaccine.

Initially, these three viruses were used in homologous prime-boost protocols, providing a significant level of protection against the chronic form of the disease in a model of toxoplasmosis in which BALB/c were challenged with the P-Br strain of the parasite [43]. However, in a different model where C57BL/6 mice were challenged with the ME49 strain, only the adenovirus encoding the SAG1 antigen showed protective properties [44]. This observation prompted us to focus our investigations in SAG1.

In the present work, we have generated a MVA encoding the SAG1 antigen (MVASAG1), to be used in a heterologous prime-boost protocol in combination with the adenovirus encoding the same antigen. Our aim was to evaluate whether the combination of two vectors could result in improved immune response and induce higher level of protection against experimental toxoplasmosis.

## Materials and Methods

### Ethics Statement

Animal housing and experimentation were strictly performed according to guidelines set forth by the Institutional Ethics Committee from the Oswaldo Cruz Foundation (FIOCRUZ), Brazil. The protocol of this study (registration number P-4/09-2) was approved by the Institutional Ethics Committee from FIOCRUZ.

### Mice

Six to eight week old female Swiss-Webster and C57BL/6 mice were obtained at the Rene Rachou Research Center (FIOCRUZ) in Belo Horizonte, Brazil.

### Parasites

The type II strain ME49 [45] was maintained by serial passage of cysts in female Swiss-Webster mice. Cysts obtained from mouse brains 60 days after infection were used for challenge of vaccinated mice. RH strain [46] was maintained by serial passages of peritoneal tachyzoites and employed in preparation of total tachyzoite lysate antigen (TLA) as previously described [47].

### Reagents

Tissue culture medium, ACK Red Cell Lysing Buffer™, anti-rabbit total IgG, anti-mouse total IgG, anti-mouse IgG1 and substrates used for ELISA and ELISPOT development were obtained from Sigma (MO, USA); anti-mouse IgG2c was purchased from Southern Biotech (AL, USA); chemiluminescent reagents and autoradiography films used for Western blot development were purchased from Amershan/GE Health Care (NJ, USA); fetal bovine serum (FBS) was obtained from GIBCO (CA, USA); the antibodies and streptoavidin-peroxidase conjugate used in ELISPOT, as well as Brefeldin A and the antibody used for intracellular staining of TNF-α were obtained from BD Biosciences (CA, USA); the antibodies used for T cell surface markers and intracellular staining of IFN-γ were purchased from eBioscience (CA, USA); the ELISA kits used for detection of cytokines secreted by spleen cells were purchased from R&D Systems Inc. (MN, USA); the MHCI-binding peptide derived from SAG1 (SP0534) used for CD8^+^ T cell stimulation [43] was synthesized at the Department of Immunology of Federal University of Minas Gerais (MG, Brazil). Rabbit anti-*T. gondii* serum used for SAG1 detection in Western-blot was produced in the department of Parasitology of Federal University of Minas Gerais.

### Recombinant MVA

The recombinant MVA encoding the SAG1 antigen was obtained by homologous recombination between the transfer vector pLW44 and the genome of the wild type MVA. The plasmid pLW44 has the green fluorescent protein (GFP) reporter gene under control of the p11 promoter (Vaccinia virus late promoter) and an expression cassette controlled by the artificial promoter mH5, which allows constitutive expression of heterologous genes [48]. After recombination, both cassettes containing the recombinant antigen and the GFP reporter are inserted in the genome of the MVA. To generate the recombinant virus, the pLW44 construct carrying the heterologous *Sag1* gene was transfected in chicken embryo fibroblasts (CEFs) previously infected (one hour earlier) with wild type MVA (at a multiplicity of infection of 1 viral particle per 10 cells). Infected/transfected CEF cultures were then incubated for three days at 37°C and 5%CO_2_. The presence of recombinant viruses in CEF cultures was confirmed by expression of green fluorescent spots in the monolayer, which could be detected in epifluorescence microscope. Supernatants and cell lysates obtained from original GFP positive cultures were used for re-infection of fresh CEF monolayers and expansion of viral seeds. After a few rounds of expansion, the viral seeds were submitted to plaque purification, to ensure elimination of wild type MVA. For this purpose, each original viral stock was submitted to limiting dilution and used to infect CEF cultures. The cultures that presented a single fluorescent plaque were collected, diluted again, and used to infect fresh CEFs. This process of limiting dilution, infection, and selection of cultures containing a single viral plaque was repeated for seven consecutive times. The single plaques obtained after the seventh round of purification were considered clones and were expanded for purification and test of SAG1 expression. Purification of viruses was performed by centrifugation at 4200×*g* in sucrose cushion (36%w/v). The recombinant viruses were identified by detection of GFP-expressing infected cells in flow cytometry, and the expression of SAG1 was evaluated by Western blot assay.

### Immunization

C57BL/6 mice received the recombinant adenovirus expressing SAG1 (AdSAG1) alone (homologous protocol) or, alternatively, in combination with the MVA expressing the same antigen (MVASAG1) in a heterologous protocol. As vaccination controls, animals received an adenovirus encoding β-galactosidase from *E. coli* (AdCTRL) [49] alone or in combination with a MVA encoding only the GFP reporter (MVACTRL). In the homologous vaccination protocol, animals received two subcutaneous doses of 10^9^ plaque-forming units (PFU) of the adenoviruses, with 6 weeks of interval between doses. In the heterologous protocol, animals received one subcutaneous prime dose with 10^9^ PFU of adenovirus followed, 4 weeks later, by an intramuscular dose of 10^7^ PFU of MVASAG1. Serum and spleen samples were obtained 14 days after the last dose of virus. In survival experiments, mice were orally challenged with 10 cysts of the ME49 strain at 14 days after the last vaccination. Mortality was followed for 50 days, and the survivors were sacrificed at the end of this period for quantification of cysts in brain.

### Spleen Cell Preparation

Spleens were collected 14 days after the last vaccination and disrupted in cell strainers to obtain single cell suspensions. Red blood cells were lysed in ACK™ buffer, and remaining spleen cells were submitted to two washes in complete medium (RPMI 1640 supplemented with 10% of FBS). Total spleen cells were plated in plain complete medium or in presence of 10 µg/ml of total tachyzoite lysate antigen (TLA) or 50 µM of a MHCI-binding synthetic peptide derived from SAG1 (SP0534). Cells were cultured for 18 to 72 hours at 37°C and 5% CO_2_. Stimulated cells and culture supernatants were used for detection of cytokines produced by T cells (IFN-γ and TNF-α).

### Detection of T Cell Cytokines

In ELISPOT assays, spleen cells (1.000.000/well) were cultured with stimuli for 24 hours in nitrocellulose-bottom microtiter plates previously coated with anti-mouse IFN-γ monoclonal antibody (clone XMG1.2, 5 mg/ml) and blocked with complete medium. After cell stimulation, plates were washed thoroughly and incubated with biotinylated anti-mouse IFN-γ antibody (clone R4-6A2, 2 mg/ml), followed by peroxidase-labeled streptoavidin (1∶200). Spots were developed with 3,3-diaminobenzidine tetrahydrochloride substrate and reactions were stopped under running water. The number of spots was determined in CTL-ImmunoSpot counter (Cellular Technology Ltd., OH, USA). For intracellular cytokine detection, total spleen cells (2–3.000.000/well) were cultured for 18 hours with stimuli. Brefeldin A (1∶1000) was added to culture in the last 4 hours of incubation. Cells were washed twice in assay buffer (PBS supplemented with 1% of bovine serum albumin). Prior staining, cells were treated with 2 µg of FcγII/III receptor blocker (purified anti-mouse CD16/CD32, clone 93) in assay buffer for 30 minutes, on ice. Then, cells were submitted to surface staining with 1 µg of anti-CD4 (PerCP-Cy5.5 conjugate, clone RM4-5) and anti-CD8γ (APC-Cy™ 7 conjugate, clone 53–6.7) diluted in assay buffer for 30 minutes, on ice. Cells were washed twice in assay buffer and fixed in 4% formaldehyde for 15 minutes at room temperature. Next, cells were permeabilized in PBS supplemented with 0.5% Tween 20, on ice, for 20 minutes. Finally, cells were stained with 1 µg of antibodies to intracellular IFN-γ (PE conjugate, clone XMG1.2) and TNF-α (PE-Cy™ 7 conjugate, clone MP6-XT22), for 30 minutes, on ice. Cells were washed twice and suspended in assay buffer. Events were acquired in a BD LSRII cytometer and analyzed in FlowJo (Three Strar Inc., OR, USA). For detection of secreted cytokines, total spleen cells (5.000.000/well) were cultured with stimuli for 72 hours. Cell-free culture supernatants were tested using commercial cytokine ELISA kits, as oriented by manufacturer. Briefly, microtiter plates were coated overnight with anti-IFN-γ capture antibody (2 µg/ml) diluted in PBS, and blocked with PBS containing 1% of bovine serum albumin (assay diluent) for 1 hour. Supernatants from spleen cell culture of vaccinated mice and an IFN-γ standard were added to plates for 2 hours. After washing plates with PBS supplemented with 0.05% Tween 20 (wash buffer), the secondary antibody was added to plates for 2 hours. Detection reagent (streptavidin-HRP conjugate) was diluted 1∶200 in assay diluent and added to previously washed plates for 20 minutes. Reactions were developed with a peroxidase substrate containing tetramethylbenzidine, stopped with 2N H_2_SO_4_ and read at 450 nm.

### Detection of Recombinant SAG1 in Cells Infected “in vitro” and Anti-SAG1 Antibodies in serum from Vaccinated Mice

For characterization of SAG1 expression, MVASAG1- or MVACTRL-infected cell lysates were run in 12% polyacrylamide gels (5 µg/lane) under denaturing conditions and transferred onto nitrocellulose membranes. In sequence, membranes were blocked in PBS supplemented with 5% skim milk, incubated with anti-*T. gondii* rabbit serum (1∶1000), washed thoroughly and then incubated with peroxidase-conjugated goat anti-rabbit total IgG (1∶3000). For detection of anti-SAG1 IgG in mouse serum, TLA (5 µg/lane) was run and transferred under same conditions as above. In sequence, TLA-loaded membrane strips were blocked, incubated with individual samples of vaccinated or control mice serum (1∶1000), washed thoroughly and then incubated with peroxidase-conjugated anti-mouse total IgG (1∶3000). All Western-blot reactions were developed with a chemiluminescent substrate and detected by exposure of membranes to autoradiography films. In ELISA assays, microtiter plates previously coated overnight at 4°C with TLA (10 µg/ml) were blocked for 2 h at 37°C with PBS supplemented with 10% FBS. Diluted serum samples (1∶50) were added for 2 h at 37°C. Detection of antibodies was performed with peroxidase-conjugated goat anti-mouse IgG1 (1∶3000) or IgG2C (1∶5000). After 45 min of incubation at 37°C, plates were washed 5 times with PBS containing 0,05% Tween 20. Reactions were developed with a peroxidase substrate containing tetramethylbenzidine, stopped with 2N H_2_SO_4_ and read at 450 nm.

### Statistical Analysis

Mortality curves of the different immunized groups after infection with ME49 strain were compared using Chi-Square test. To compare the number of brain cysts we used t test of Mann-Whitney; to compare IgG titer in serum we used paired t test. To compare the number of spots, percentage of cytokine secreting cells and levels of secreted cytokines we used two-way ANOVA with Bonferroni’s post-test for multiple comparison. All tests were performed in GraphPad Prism, version 5.0, (GraphPad Softwares Inc., La Jolla, USA), and differences were considered statistically significant when *p*<0.05.

## Results

### Generation of a Recombinant MVA Expressing the SAG1 Protein from *T. gondii*

**Figure 1:**
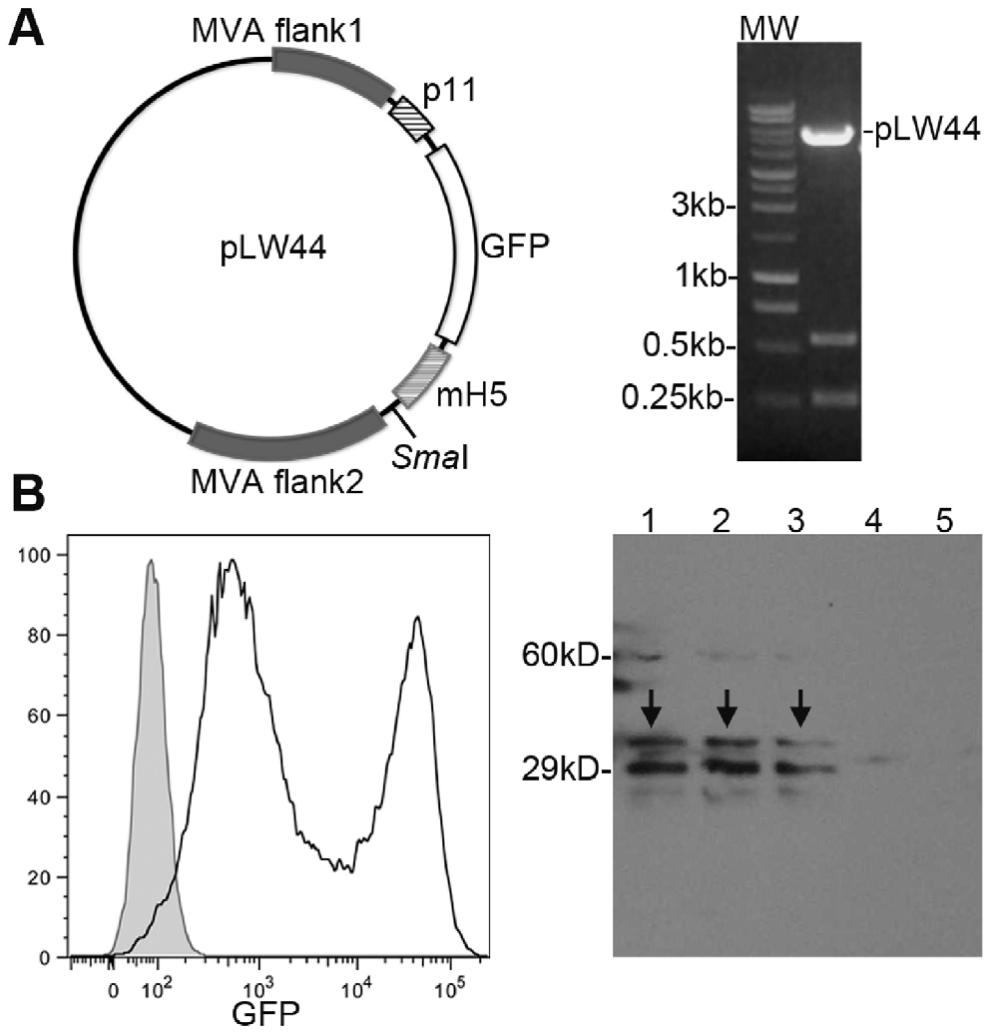
Generation of the recombinant MVA expressing the Surface Antigen 1 (SAG1) of *T.gondii*. A, left: plasmid pLW44 was used as shuttle vector for recombination with wild-type MVA genome and insertion of the *Sag1* coding sequence into the virus. The *Sag1* coding sequence was subcloned in the *Sma*I site of plasmid pLW44 under control of the promoter mH5 and flanked by two MVA sequences. A, right: digestion of the construct pLW44-SAG1 to verify the correct orientation of the transgene in relation to promoter. B, left: flow cytometry analysis of cells infected with MVASAG1. The presence of recombinant MVA virus is indicated by expression of green fluorescence (FITC channel). In the histogram, gray filled area corresponds to non-infected cells and black line corresponds to MVASAG1 infected cells. B, right: expression of SAG1 in MVASAG1 infected cells. In the Western blot, infected cell lysates were tested against serum from rabbit chronically infected with *T. gondii* RH strain. Lines 1–3: lysates of cells infected with three different clones of MVASAG1. Line 4: lysate of cells infected with a control MVA expressing only GFP (MVACTRL). Line 5: lysate of non-infected cells. The arrows indicated the bands corresponding to SAG1.

The recombinant MVASAG1 was generated by homologous intracellular recombination between the plasmid pLW44 encoding the *Sag1* gene and the genome from a wild type MVA. The sequence of *Sag1* gene was excised from plasmid pAdSAG1 [43] using *Bgl*II and *Hind*III endonucleases. The staggered ends were filled in with T4 DNA polymerase and the blunt-ended fragments inserted into the *Sma*I site of pLW44 plasmid (figure 1A, left). The construct containing *Sag1* in the correct orientation (Figure 1A, right) was transfected into CEFs previously infected with the wild type MVA. Positive recombination between pLW44-SAG1 and MVA, as well as generation of new viruses was confirmed by expression of the GFP reporter in infected/transfected cells (Figure 1B, left). Newly obtained recombinant viruses were submitted to seven rounds of selection, to eliminate contamination with the wild type virus. Expression of the SAG1 protein by the recombinant viruses was confirmed by Western blot assay with extracts of infected CEFs, as showed in figure 1B (right).

### Humoral Immune Response after Vaccination with Recombinant Viruses Encoding SAG1 Protein from *T. gondii*

**Figure 2:**
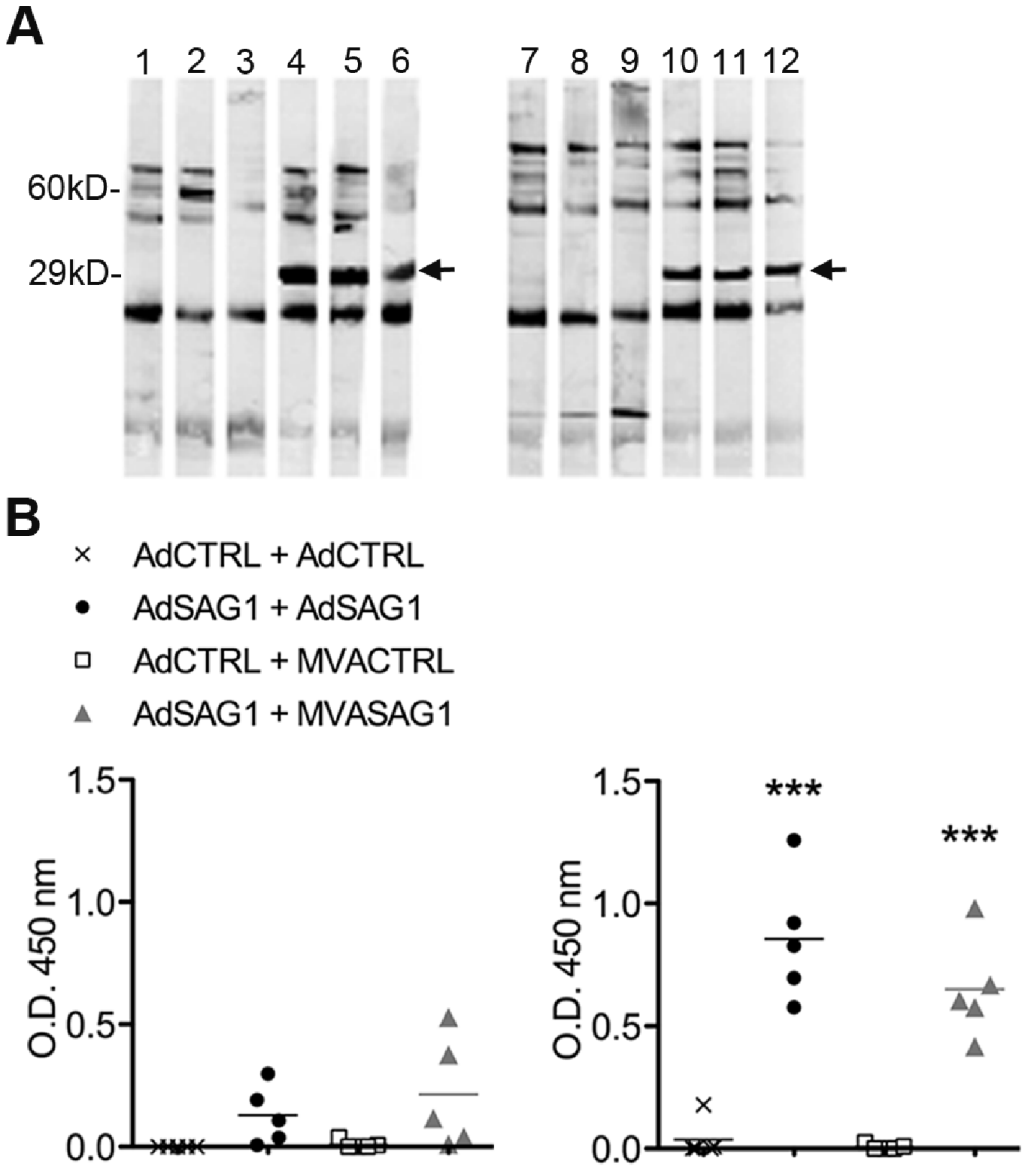
Analysis of the antibody response in C57BL/6 mice vaccinated with viral vectors expressing the antigen SAG1 of *T.gondii*. Animals were vaccinated according to homologous (Adeno+Adeno) or heterologous (Adeno+MVA) protocol, as described in table 1. Serum samples were obtained two weeks after last vaccination, and tested individually for presence of anti-SAG1 specific antibodies. A, sera from three animals/group were tested individually in Western blot against *T. gondii* lysate (TLA). Lanes 1 to 6: serum from homologous-vaccinated groups (1 to 3 AdCTRL+AdCTRL, 4 to 6 AdSAG1+ AdSAG1). Lanes 7 to 12: serum from heterologous-vaccinated groups (7 to 9 AdCTRL+MVACTRL, 10 to 12 AdSAG1+ MVASAG1). Arrows indicate serum reactivity with SAG1. B, serum samples (5 animals/group) were tested in ELISA for presence of IgG1 (left) and IgG2c (right) antibodies against TLA. Plots show individual mouse (symbols) and group mean (line). Asterisks indicate significant difference between SAG1-vaccinated mice and counterpart control-vaccinated group (***p<0.001). Data obtained in one experiment. The experiment was performed two times, independently.

In order to verity if the immunization with vectors encoding the SAG1 antigen induced adequate *in vivo* expression of the protein, we evaluated the presence of specific anti-SAG1 antibodies in mouse serum samples after vaccination. C57BL/6 mice were immunized according to a homologous protocol consisting of two doses of adenovirus or, alternatively, were submitted to a heterologous protocol, with one prime dose of adenovirus followed by one dose of MVA (table 1). Two weeks after the last viral dose, the serum samples were tested in Western blot, using total tachyzoite lysate (TLA) as antigen. Figure 2A shows that sera from all SAG1-vaccinated mice reacted with one band around 30 kD corresponding to SAG1. The serum samples were also tested in ELISA assays, which showed presence of different classes of anti-SAG1 IgG antibodies, namely IgG1 and IgG2c, in vaccinated mice. The levels of both IgG subclasses were comparable between the two vaccination protocols. It is known that production of IgG subclasses is driven by cytokines secreted during cellular immune responses. Therefore, the presence of IgG1 and IgG2c suggests indirectly that the both vaccination protocols also promoted activation of cell-mediated response.

**Table 1:** Homologous and heterologous vaccination protocols.

	Prime				Boost		
Protocol	Vector	Dose (PFU)	Route	Interval (weeks)	Vector	Dose	Route
Homologous	AdSAG1	10^9^	s.c.	6	AdSAG1	10^9^	s.c.
	AdCTRL	10^9^	s.c.	6	AdCTRL	10^9^	s.c.
Heterologous	AdSAG1	10^9^	s.c.	4	MVASAG1	10^7^	i.m.
	AdCTRL	10^9^	s.c.	4	MVACTRL	10^7^	i.m.

s.c.: subcutaneous; i.m.: intramuscular; PFU: plaque-forming units (infective units).

### Cellular Immune Response after Vaccination with Recombinant Viruses Encoding the SAG1 Protein from *T. gondii*

**Figure 3:**
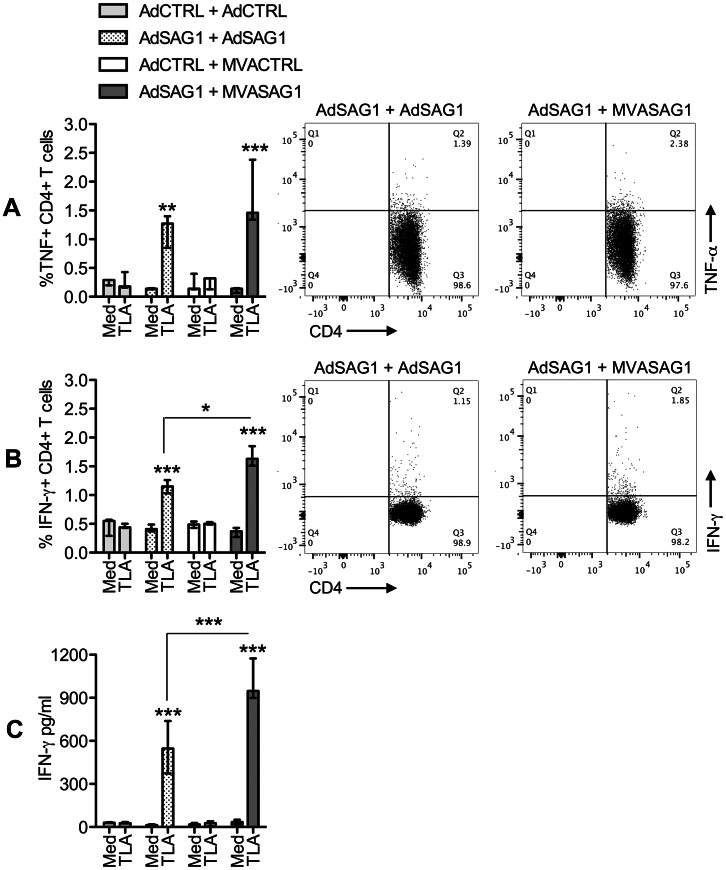
Analysis of CD4^+^ T cell response in C57BL/6 mice vaccinated with viral vectors expressing the SAG1 protein of *T.gondii*. Total spleen cells were obtained two weeks after vaccination and cultured in plain complete medium (Med) or with TLA. The stimulated cells were stained for CD4^+^ T cell markers and intracellular TNF-α and IFN-γ and analyzed in flow cytometry. The supernatant of stimulated cells were tested for presence of secreted cytokines in ELISA. A, percentage of TNF-α-producing cells inside the CD4^+^ T cell gate. B, percentage of IFN-γ-producing cells inside the CD4^+^ T cell gate. In A and B, the bar graphs on the left show the median±range of 3 animals tested individually, whereas the plots on the right show the response to TLA of one representative animal of each SAG1-vaccinated group. C, levels of IFN-γ in spleen culture supernatants. The bar graphs show the median±range of 3 animals tested individually. Asterisks indicate significant differences between groups (*p<0.05; **p<0.01; ***p<0.001). Data obtained in one experiment. The experiment was performed two times, independently.

**Figure 4:**
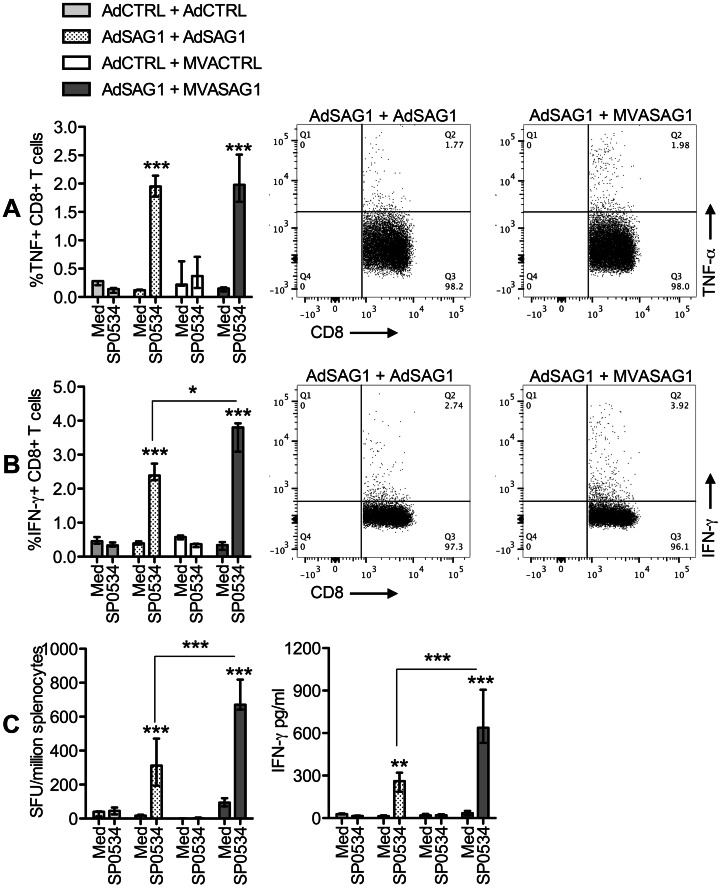
Analysis of CD8^+^ T cell response in C57BL/6 mice vaccinated with viral vectors expressing the SAG1 protein of *T.gondii*. Total spleen cells were obtained two weeks after vaccination and cultured in plain complete medium (Med) or with the MHCI-restricted peptide SP0534, derived from SAG1. The stimulated cells were stained for CD8^+^ T cell markers and intracellular TNF-α and IFN-γ and analyzed in flow cytometry. Alternatively, spleen cells were tested in an IFN-γ ELISPOT assay. The supernatant of stimulated cells were tested for presence of secreted cytokines in ELISA. A, percentage of TNF-α-producing cells in the CD8^+^ T gate. B, percentage of IFN-γ-producing cells in the CD8^+^ T gate. In A and B, the bar graphs on the left show the median±range of 3 animals tested individually, whereas the plots on the right show the response to SP0534 of one representative animal of each SAG1-vaccinated group. C, left, frequency of IFN-γ secreting units in total spleen cells, measured in ELISPOT. C, right, levels of IFN-γ in spleen culture supernatants. The bar graphs show the median±range of 3 animals tested individually. Asterisks indicate significant differences between groups (*p<0.05; ***p<0.01, ***p<0.001). Data obtained in one experiment. The experiment was performed two times, independently.

Two weeks after the last viral dose, splenocytes were obtained from immunized mice and submitted to stimulation with *T. gondii* antigens. Then, we evaluated the frequencies of T cells producing IFN-γ and TNF-α, as described in the Methods section and figure S1. Figure 3 shows that, upon stimulation with TLA, spleens from animals that received the viruses encoding SAG1 presented higher frequencies of CD4^+^ T cells that produced TNF-α (figure 3A) and IFN-γ (figure 3B) in comparison to control-vaccinated mice. We observed that the heterologous-vaccinated mice tended to develop higher frequencies of TLA-responding CD4^+^ T cells, although, in the case of TNF-α producing lymphocytes, the difference between protocols did not reach the level of statistical significance. The discrete increase in the number of IFN-γ producing CD4^+^ T lymphocytes was reflected in a modest but significant increment in the levels of secreted IFN-γ in supernatants of spleen cell cultures from heterologous-vaccinated animals (figure 3C). The amount TNF-α in cell supernatants was below detection level of the assay in all groups. Analysis of the specific CD8^+^ T cell populations elicited after vaccination yielded similar findings (figure 4, figure S2). Heterologous-vaccinated tended to develop higher numbers of TNF-α (figure 4A) and IFN-γ (figure 4B) producing CD8^+^ T cells in response to stimulation with the MHCI-restricted SAG1 peptide SP0534. The IFN-γ ELISPOT assay (figure 4C, left) confirmed the increased frequency of IFN-γ secreting specific cells in heterologous-vaccinated spleens, which was reflected in higher levels of secreted IFN-γ in spleen cultures from this group (figure 4C, right).

### Protection Induced by Vaccination with Recombinant Virus Encoding SAG1 Protein from *T. gondii*

**Figure 5:**
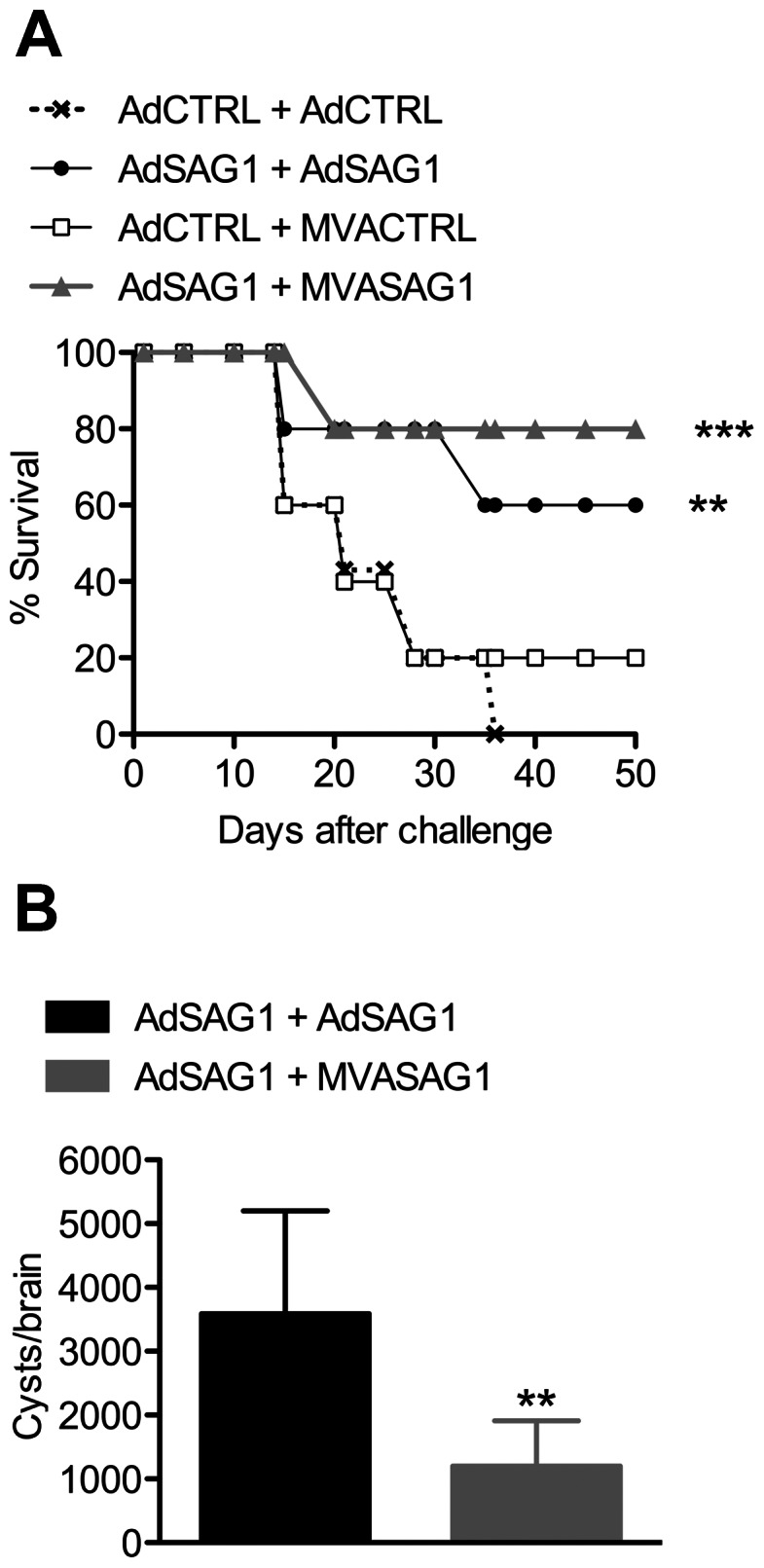
Challenge of vaccinated mice with ME49 strain of *T.gondii*. C57BL/6 mice (10 per group) were immunized with two doses of recombinant viruses according to the homologous or heterologous protocol. Two weeks later, animals were challenged with an oral dose of 10 cysts of ME49. A, mice were followed for survival for 50 days after infection. B, the animals that survived past 50 days of infection (6 animals in group AdSAG1+ AdSAG1; 8 animals in group AdSAG1+ MVASAG1) were sacrificed for counting the number of cysts in brain. Bars represent mean±SD. In A, asterisks indicate significant differences between SAG1-vaccinated and control-vaccinated groups. In B, asterisks indicate difference between the homologous-vaccinated and heterologous-vaccinated group (**p<0.01; ***p<0.001). Data obtained in one experiment. The experiment was performed two times, independently.

To evaluate the protection conferred by each vaccination protocol, we challenged the mice with the ME49 strain of *T. gondii*. As observed in other models of recombinant vaccines, which showed that protection is achieved only with multiple doses of the recombinant immunogen, the administration of a single dose of either AdSAG1 or MVASAG1 did not prevent mortality (figure S3). In the case of mice immunized with two viral doses, we verified a tendency of higher survival of animals from heterologous protocol comparing to homologous protocol (figure 5A). Also, we evaluated the number of cysts in brains of surviving animals. As shown in figure 5B, we observed that mice from heterologous vaccination groups developed a significantly lower (*p*<0.01) number of brain cysts in comparison to animals from homologous vaccination groups.

## Discussion

Recombinant vaccines have a great potential for future utilization for prevention and therapy of diseases caused by intracellular parasites, including *T. gondii*. The success of a recombinant vaccine depends on many factors, including identification and selection of those parasite components targeted by host immune system and the design of powerful vectors that can induce efficient “in vivo” expression of those antigens. Another key point is characterization of innate immune mechanisms and adjuvants that regulate the activation of the acquired immune response and development of memory after vaccination. Finally, of equal importance is the development of vaccination protocols that ensure that the host immune system will be exposed to antigens and adjuvants in the adequate amount and for the right length of time.

Our strategy for development of an anti-toxoplasmosis recombinant vaccine has been the generation of viral vectors expressing *T. gondii* antigens. Most of our work has centered in the surface protein SAG1, which is expressed in tachyzoites, and mediates adhesion of the parasite to the surface of the host cell prior invasion [40], [50]. SAG1 has epitopes that are recognized by mice experimentally inoculated with *T. gondii* and by humans naturally infected with the parasite [51]. These characteristics make SAG1 a promising candidate for development of a vaccine. In fact, in studies from other groups, SAG1 administered to mice in different forms and protocols was able to protect against a variety of *T. gondii* strains [52]–[57].

Regarding the vaccine vectors used for SAG1 expression, we started our work with the replication-deficient Type 5 Human Adenovirus. Besides being highly efficient for transgene expression “in vivo” and safe for administration, adenoviral vectors also have intrinsic adjuvant properties, being capable of activating innate immune response via TLR [58], [59] and NLR [60] receptors. Consequently, adenoviral vectors are excellent for inducing Th1-biased cellular responses, with activation of CD4^+^ T and CD8^+^ T cells that are capable of producing IFN-γ – a type of acquired response that is essential for protection against *T. gondii*. Using the AdSAG1 in a homologous protocol we obtained partial protection against cyst formation in a model of chronic Toxoplasmosis caused by the P-Br strain in a BALB/c mice [43]. The same vaccine was able to reduce acute mortality and reduce cyst formation in C57BL/6 mice after challenge with ME49 strain. Protection was correlated with the capacity of AdSAG1 in activating CD8^+^ T cell-mediated responses, in a mechanism dependent of MyD88 innate signaling and IL-12 production [44].We are now addressing the improvement of the vaccination strategy to enhance the levels of protection. In that sense, one of the major limitations for the viral vectored vaccines is the existence of anti-vector immunity, because it can reduce the efficiency of the immunization [61]. Anti-vector immunity can be induced by natural exposition of hosts to the wild type viruses that are used as base for construction of the vectors. Besides that, anti-vector blocking responses are induced during the course of vaccination with the recombinant viruses.

One important anti-viral mechanism is production of neutralizing antibodies, which can prevent the efficient transduction of host cells by the recombinant viruses and, consequently, impair antigen expression and presentation “in vivo” [27], [62], [63]. Moreover, the immune response against viral epitopes can trigger quick elimination of transduced cells, which could result in weak immunogenicity and low protective profile of some adenovirus-based vaccination protocols [64]. It was demonstrated that transfer of serum from animals previously exposed to adenovirus to non-immune mice limits the target gene expression and that the transfer of CD8^+^ T cells limits the immune response against the gene carried by the viral vector [65]–[67]. Also, pre-existing anti-adenovirus specific T cells were accounted for decreased cellular immune response observed in an anti-HIV vaccination trial conducted in humans, using adenovirus as vaccine vector [68]. These observations suggest that the level of pre-existing immunity against adenovirus may be one of the underlying causes of variation in efficacy in different vaccination models that used those viruses [69].

Some strategies have been employed to avert the anti-adenovirus immunity, including the use of rare human serotypes or non-human serotypes for development of vaccination vectors [70], [71]. Other approach is the introduction of modifications in surface antigens and use of polymers able to mask the viral surface from neutralizing antibodies [72], [73]. Although these methodologies may reduce the effect of pre-existing antibodies generated by natural exposition of hosts to wild type viruses, they cannot prevent the induction of anti-vector immunity during vaccination. In this case, the approach to avoid anti-vector immunity is the combination of various vectors carrying the same antigen, in the so-called heterologous protocols [29], [74].

In the last years, MVA has become a very popular recombinant viral vector for the development of immunization protocols. This vector is capable to activate both humoral and cellular immune responses against the heterologous gene. Also, different studies have proved that MVA is a safe vector, for both humans and animals [38], [75]. MVA has been frequently indicated as the vector of choice to reinforce the initial immune response generated after prime with DNA or adenoviruses [76]–[78]. Taking the previous observations into consideration, we chose to generate a recombinant MVA encoding the antigen SAG1 from *T. gondii*, to be used with adenoviruses in prime-boost immunization protocols. The MVASAG1 vector was capable to induce high levels of expression of the recombinant protein SAG1 *in vitro* and showed stability after several passages in CEFs (data not shown).

Here, we have compared a homologous vaccination protocol with two doses of AdSAG1 and the heterologous protocol with one dose of AdSAG1 followed by MVASAG1. We observed that both protocols were able to induce protection against challenge. Although the protection against mortality was comparable between the protocols, the heterologous vaccination promoted significant reduction in parasite burden in brain when compared to the homologous immunization. This difference does not seem to be related to the vaccine-induced humoral response, as all SAG1-immunized groups developed similar levels of IgG1 and IgG2c antibodies. This observation is consistent with another study showing that B cell-deficient mice vaccinated with an attenuated strain of *T. gondii* were able to control parasite burden after oral challenge with ME49 as efficiently as the wild type mice [7].

On the other hand, it is well established that protection against cyst formation is primarily dependent on strong cell-mediated immunity [79]–[81]. Nevertheless, the analysis of the T cell responses showed only a modest difference between the two protocols, at least in terms of frequency of T CD4^+^ and T CD8^+^ cells producing IFN-γ or TNF-α. This indicates that other important aspects of the T cell function that were not evaluated here may be differentially activated by the vaccination protocols and may account for the differences in the protection against cysts. One such aspect is the polyfunctionality of the T cells, or the capacity of producing multiple cytokines at the same time. Several vaccination studies have correlated polyfunctionality with the improvement of the protection against infectious challenge [82]–[84]. Reyes-Sandoval and co-workers, for example, demonstrated in a mice model of *P. berghei* that adenovirus-MVA heterologous vaccination protocols resulted in activation of polyfunctional T cells that produced IFN-γ, TNF-α and IL-2, which was not observed in the homologous vaccination [85]. These reports suggest that a thorough evaluation of the efficacy of a vaccination protocol should involve not only the measurement of the potency of the response, but also of its quality. Although we were not able to evaluate the induction of polyfunctional T cells in our model, we speculate they may be induced more efficiently by adenovirus-MVA vaccination, resulting in enhanced capacity to control the load of brain cysts in this group.

Another important aspect of the T cell function is the elimination of parasites and infected cells by cytotoxic CD8^+^ T lymphocytes. Some studies have shown that CTLs are involved in cyst control during *T. gondii* infection, in a mechanism mediated by perforin [86]. We attempted to develop an *in vivo* cytotoxicity assay, in which SAG1-vaccinated mice were inoculated with target cells coated with the SAG1 epitope TPTENHFTL (SP0534). The initial assays, however, failed to detect any CTL activity, in both homologous and heterologous protocol (data not shown). Further investigation is necessary to determine the status of CTL activity in our vaccination model.

Finally, one advantage of the heterologous protocols, as demonstrated in other vaccination models, is the enhanced capability of inducing long-lasting T cell memory [87]. Although we have not investigated the memory T cell responses in our study, the data showing better control of development of brain cysts in heterologous vaccinated mice during the chronic phase of toxoplasmosis, which is dependent in long-lasting T cells, is an indicative of superior memory induction by heterologous protocol.

In summary, our results suggest that the combination of adenovirus-MVA vectors expressing SAG1 is capable to improve the protection against cyst formation during chronic toxoplasmosis, in comparison to a homologous protocol using two doses of adenoviruses encoding SAG1 antigen from *T. gondii.* This enhanced protection was not related to increase in the magnitude of the T cell response against SAG1. The real mechanisms behind the improvement of cyst control need further investigation.

## Supporting Information

Figure S1 **Gating strategy for analysis of cytokine production in CD4^+^ T cells.** A, single cells were gated in the total live spleen cell population using FCS-A x FSC-H parameters. The CD4^+^ cells and CD8^+^ cells were gated in the single cell population by plotting PerCP-Cy5.5 fluorescence (CD4 stain) against APC-Cy™ 7 (CD8 stain) parameters. B, IFN-γ producing CD4^+^ T cells were identified by plotting PerCP-Cy5.5 (CD4 stain) against PE (IFN-γ stain). C, TNF-α producing CD4^+^ T cells were identified by plotting PerCP-Cy5.5 (CD4 stain) against PE-Cy™ 7 (TNF-α stain).
(TIFF)

Figure S2 **Analysis of cytokine production in CD8^+^ T cells. The CD8^+^ cells were gated in the total live spleen cells as shown in figure S1.** A, The IFN-γ-producing CD8^+^ T cells were identified by plotting APC-Cy™ 7 (CD8 stain) against PE (IFN-γ stain). TNF-α producing CD8^+^ T cells were identified by plotting APC-Cy™ 7 (CD8 stain) against PE-Cy™ 7 (TNF-α stain).
(TIFF)

Figure S3 **Challenge of animals immunized with a single dose of viral vectors encoding SAG1. C57BL/6 mice (10 per group) received one dose of 10^9^** **p.f.u. of adenovirus (control or SAG1) or one dose of 10^7^** **p.f.u. of MVA (control or SAG1).** Two weeks after challenge, the animals received one oral dose of 10 cysts of the ME49 strain of *T. gondii*.
(TIFF)
