# Supplementary figures and images for: Recombinant Vaccines against T. gondii: Comparison between Homologous and Heterologous Vaccination Protocols Using Two Viral Vectors Expressing SAG1
Source: PLoS One. 2013 May 15;8(5):e63201. doi: 10.1371/journal.pone.0063201 (PMC3654925; doi:10.1371/journal.pone.0063201)

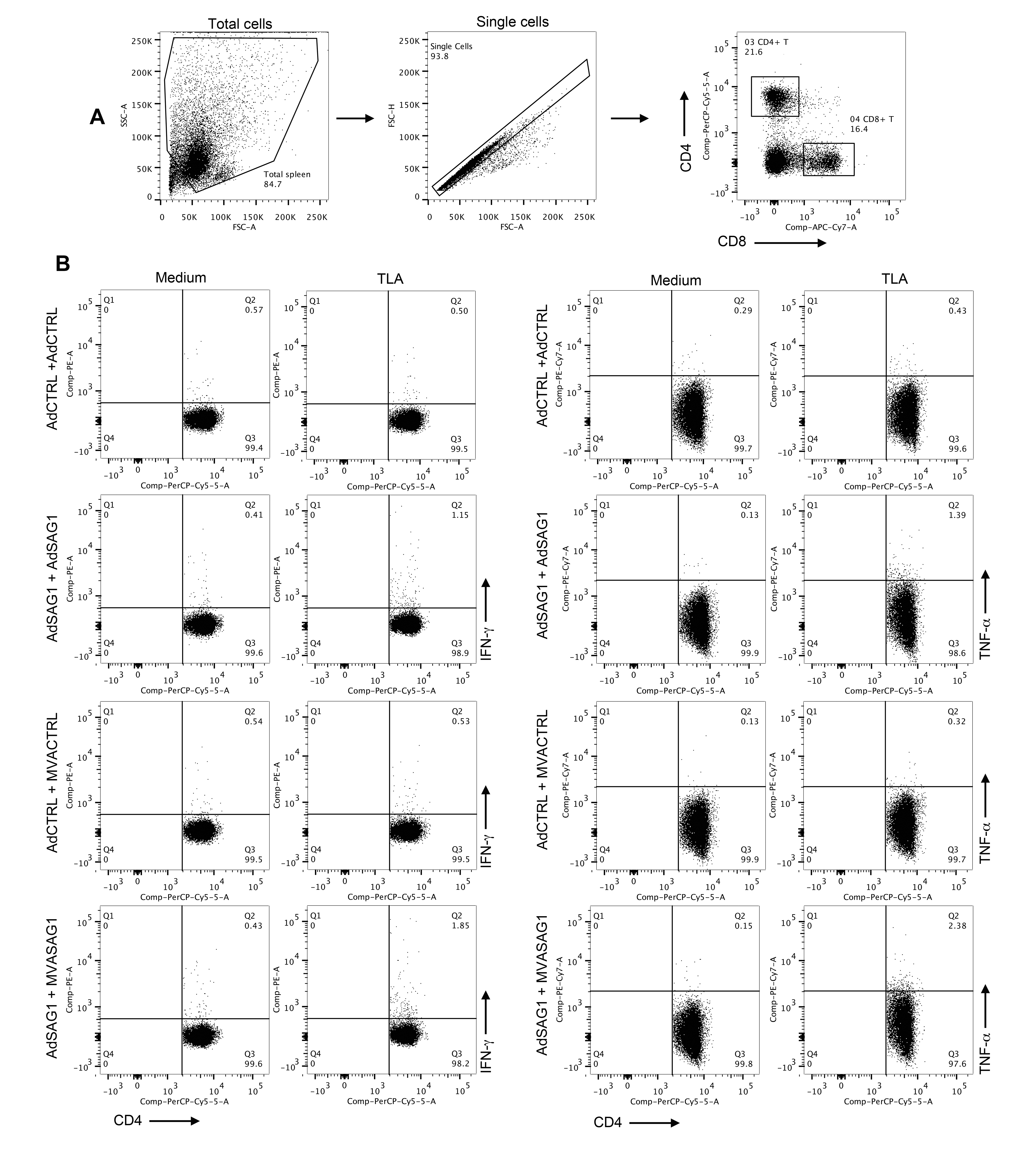

Supplement: Figure S1 — Gating strategy for analysis of cytokine production in CD4+ T cells. A, single cells were gated in the total live spleen cell population using FCS-A x FSC-H parameters. The CD4+ cells and CD8+ cells were gated in the single cell population by plotting PerCP-Cy5.5 fluorescence (CD4 stain) against APC-Cy™ 7 (CD8 stain) parameters. B, IFN-γ producing CD4+ T cells were identified by plotting PerCP-Cy5.5 (CD4 stain) against PE (IFN-γ stain). C, TNF-α producing CD4+ T cells were identified by plotting PerCP-Cy5.5 (CD4 stain) against PE-Cy™ 7 (TNF-α stain). (TIFF) [file pone.0063201.s001.tif]

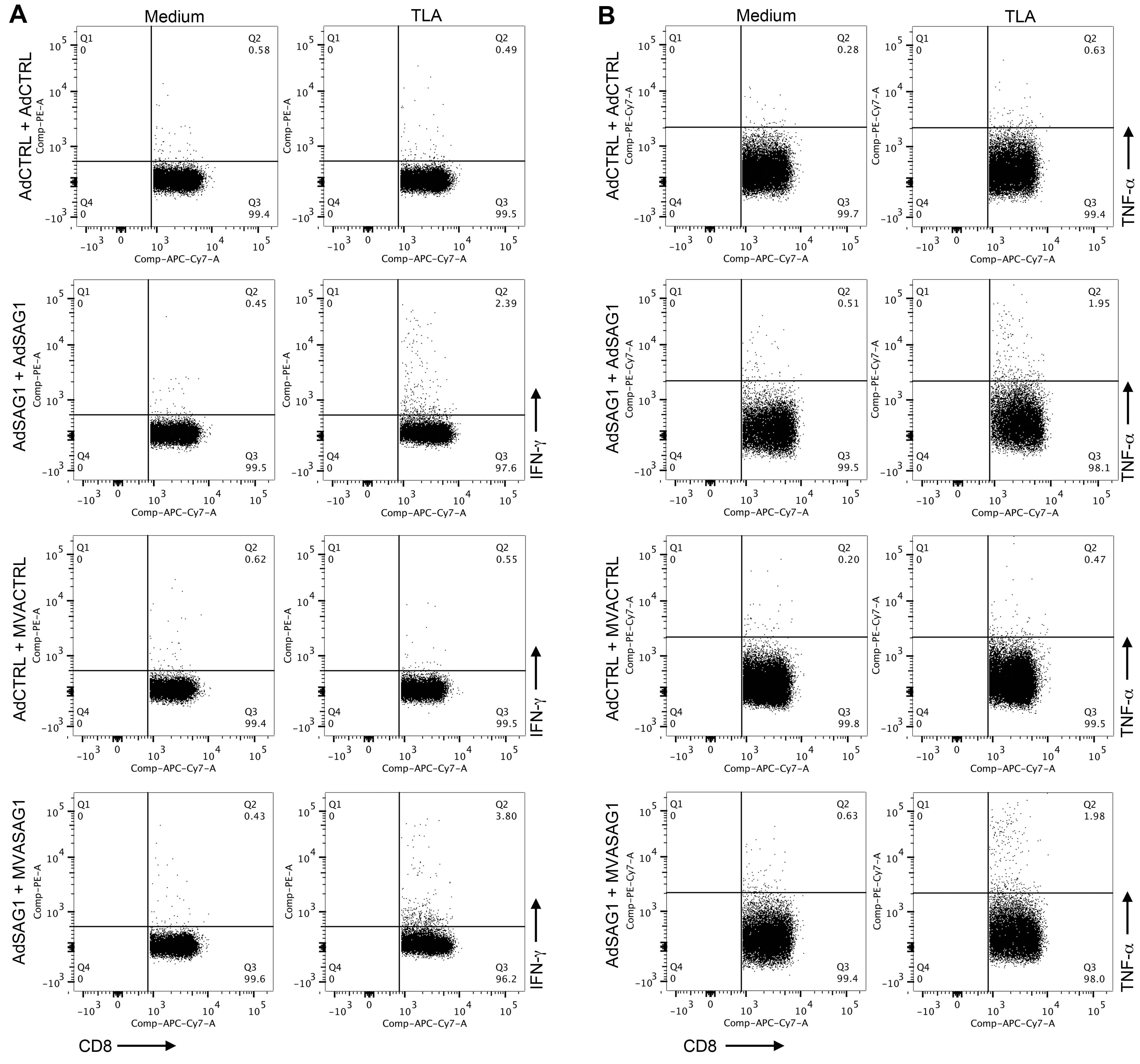

Supplement: Figure S2 — Analysis of cytokine production in CD8+ T cells. The CD8+ cells were gated in the total live spleen cells as shown in figure S1. A, The IFN-γ-producing CD8+ T cells were identified by plotting APC-Cy™ 7 (CD8 stain) against PE (IFN-γ stain). TNF-α producing CD8+ T cells were identified by plotting APC-Cy™ 7 (CD8 stain) against PE-Cy™ 7 (TNF-α stain). (TIFF) [file pone.0063201.s002.tif]

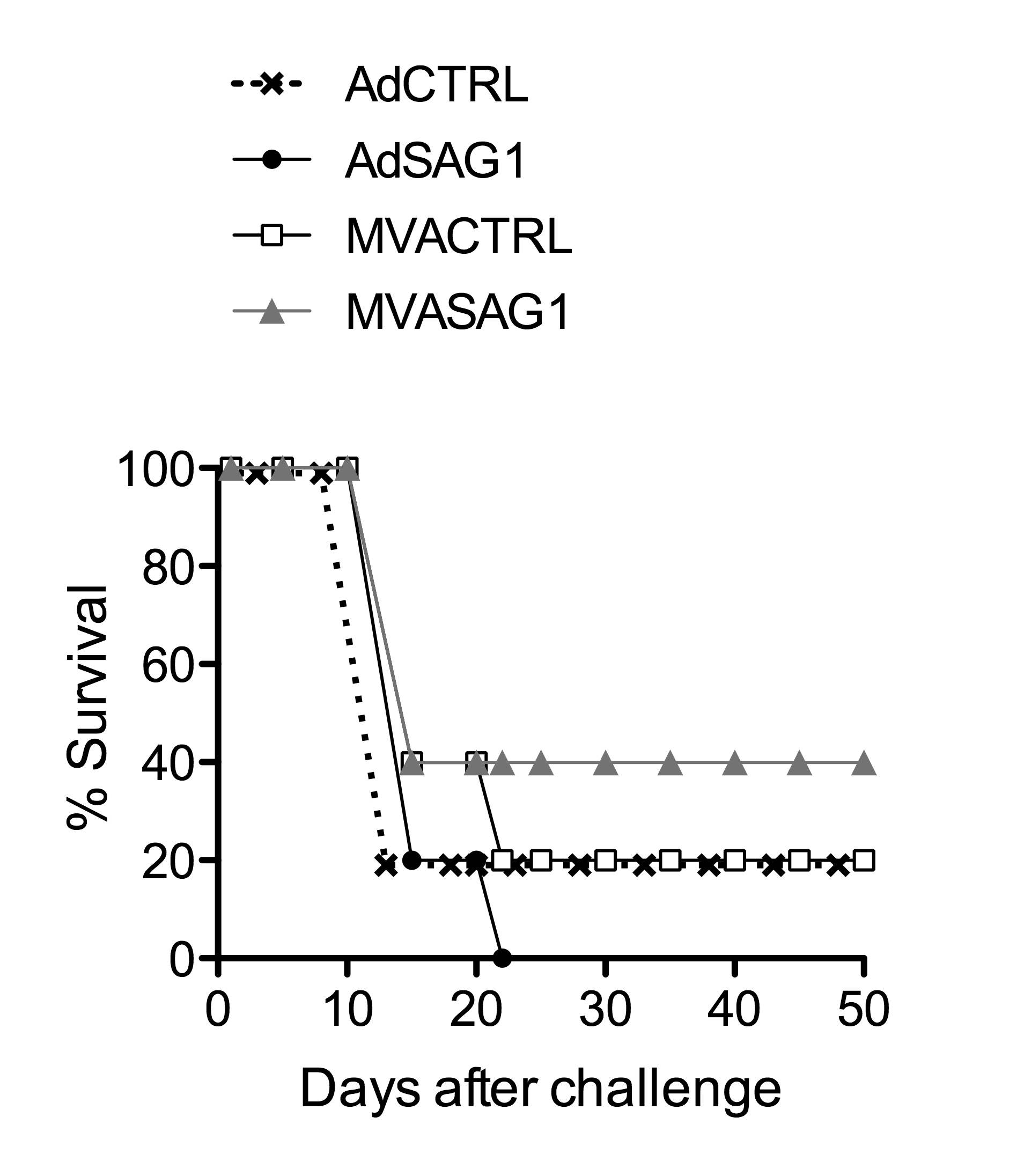

Supplement: Figure S3 — Challenge of animals immunized with a single dose of viral vectors encoding SAG1. C57BL/6 mice (10 per group) received one dose of 109 p.f.u. of adenovirus (control or SAG1) or one dose of 107 p.f.u. of MVA (control or SAG1). Two weeks after challenge, the animals received one oral dose of 10 cysts of the ME49 strain of T. gondii. (TIFF) [file pone.0063201.s003.tif]
